# A Case of Gunshot Wound of the Stomach, with Three Perforations of the Same

**Published:** 1896-07

**Authors:** L. A. Woodson

**Affiliations:** Palton, Ala.


### A CASE OF GUNSHOT WOUND OF THE STOMACH WITH THREE PERFORATIONS OF SAME.

BY DR. L. A. WOODSON, M. D., PALTON, ALA.

I suppose that in no department of medical science have more beneficent and salutary advances been made than that of surgery, and these advances, reckoning them from their present state of almost perfection, date back but a little more than a decade. In the retrospection of a varied experience of some thirty-five years in the practice of medicine, four years of which were passed as surgeon in the Confederate Army, I fail to recall a single case of multiple perforations of the stomach or bowels, which did not have a fatal termination, until up to some ten or fifteen years ago. I have no doubt that there were some cases of the kind that did recover, but they were rare exceptions to the general rule; and the patient might be said to have struggled through the shadow of death into life, unaided except by the *vis medicatrix naturae*, in so far as any systematic and scientific plan of procedure was followed in his treatment. But at the present time no wound of the character indicated needs, it seems, to be regarded as necessarily fatal.

The observations above made were suggested by a case which occurred in my practice a few weeks since, and one which I consider of sufficient interest to place upon record.

On the 18th of March last I was summoned at midnight to attend a negro man about 25 years of age, who, the messenger informed me, had been desperately wounded by a pistol shot inflicted by another negro with whom he was engaged in a fight. On arriving at the home of the patient I found him suffering acutely from the effects of the wound. The ball from a 38-calibre Smith & Wesson pistol had entered the left side just below the apex of the heart, and the probe, influenced by the gentlest manipulation, clearly indicated that the ball had penetrated the cavity. The track of the ball trended slightly downward, and, the course, from left to right. The man was suffering greatly from shock, the pulse being 120, very weak and unsteady. I also found that he had vomited a considerable quantity of blood, which his friends informed me was almost black in color. From this latter symptom and the direction of the track of the ball, I felt satisfied that the stomach had been perforated. The lateness of the hour, the imperfect light, and my unwillingness to proceed unaided in a case of such gravity, determined me to postpone surgical interference until the following day, and convinced by considerable experience in cases of kindred character, that such interference gave promise of the only rational hope for his recovery. I therefore gave the man a half a grain of sulphate of morphine to relieve pain and tranquillize his nervous system, and left him for the night. The next day I sent for my son, Dr. J. L. Woodson, to perform the operation. He did not arrive until 4 o'clock P. M. The patient at this time was in a most critical condition, and so enormously swollen over the whole abdominal region from internal hemorrhage as to tilt upward at an angle of about forty-five degrees the ensiform appendage and costal cartilages. Blood was oozing from the external wound, and the man complained of considerable difficulty of breathing, due both to hemorrhage and upward pressure of the blood against the diaphragm. His pulse was very weak, and 125 to the minute. In addition to all this, he had at intervals during the morning vomited considerable quantities of blood mixed with fæcal matter. Finding the man in such a desperate condition, my son hesitated as to the expediency of performing so grave an operation as laparotomy, fearing lest the man should die before its completion. But feeling absolutely certain that without such an operation he could not survive many hours, he decided to perform it, though entertaining but little hope of any permanent benefit resulting.

Having determined upon this course of procedure we laid him upon the table, scrubbed well with soap and water the whole anterior surface of the abdomen, and after wiping him quite dry repeated the process with a solution of bichloride of mercury. We then gave him about three ounces of whisky, which he retained. As we judged that considerable time would be consumed in the operation we decided upon ether as the anæsthetic. In about seven or eight minutes from the commencement of its administration he was thoroughly under its influence. My son, having selected the knife to be used from a dish containing all of the instruments to be employed in the operation, immersed in a hot solution of the bichloride, made the primary incision, extending from the ensiform appendage to the umbilicus. When the incision had been carried to the requisite depth, a grooved director was entered at its lower extremity and the peritoneal cavity opened up to the sternum. No sooner had this been done than , under the powerful pressure exerted by the effused blood which filled the cavity, both the stomach and intestines were forced through the opening, the latter were protected from chills and injury by envelopment in towels, saturated with warm salt water. Bent spatulæ were then applied to the lips of the wound, and under steady traction it was so widened as to allow the stomach to be lifted as high through the opening as its internal attachments would with safety permit.

The first discovery made upon examination was that the ball had entered its cavity at the cardiac end, and in so doing had severed the gastro-epiploica sinistra artery. This artery was bleeding quite freely, and was at once secured by the finest carbolized silk ligature. The gut ligature would have been used, but we did not possess it at the time. The wound in the stomach was then sewed up with the same kind of ligature, employing for the closure of this wound the Lembert suture. The stomach was now turned as far over to the left side as possible in order to inspect its under surface. When this was done we found that the ball had emerged to the right of the esophageal opening, and then making another perforating wound an inch long in the pyloric end of the same. These wounds were also closed in the same manner as the first. The ultimate destination of the ball is a matter of conjecture, as the condition of the patient and the length of time already consumed in the operation forbade the idea of further exploration, but the direction of its course, together with the large quantities of bile ejected during the night preceding the operation, would indicate that its final lodgment was in the liver.

Having now done all for the man that a prudent regard for his safety seemed to warrant, we turned him upon his side in order to allow as much of the effused blood as would do so to escape, since the quantity contained in the cavity rendered its absorption by sponges both difficult and tedious. For the thorough removal of that which remained the sponges were used, and the peritoneal cavity flushed with warm water holding in solution the bichloride of mecrury, 1 to 3,000. This being done, the stomach and bowels were carefully replaced, the drainage tubes adjusted, the corresponding surfaces of the wound accurately approximated and secured by deep-seated sutures of carbolized silk; iodoform was freely dusted over the entire length of the wound, strong adhesive strips, two inches in width and thirteen inches in length, were applied transversely across the line of incision to relieve undue tension upon the stitches; antiseptic gauze was laid over these strips, then a layer of absorbent cotton and a wide rubber bandage encircling the body from the sternum to a little below the umbilicus completed the dressing.

The time consumed in the operation, including the dressing, was forty minutes. After having been aroused to consciousness, we gave him a hypodermic injection of strychnia, and cautioned his nurses to allow no food or drink to be taken into the stomach, but to be given by injection into the rectum.

On visiting him the next morning, I was astonished to find him entirely free of fever, his temperature was only 98½ degrees and his pulse 85, and at no time thereafter did either attain a higher elevation. His food up to the 26th of March, eight days after the operation, as well as all the drinks allowed, were injected into the rectum. Five days after the operation a dose of sulphate of magnesia was given, and three copious discharges followed its administration. The dressings were all removed on the eighth day, showing the wound nearly healed through its entire length, by first intention. Several of the stitches were removed at this time; the drainage tube had already been removed on the third day. Satisfied as to the condition of the wound the various dressings were reapplied as in the first instance, and the wound was examined again on the 30th of March. This inspection showed the wound entirely healed. All of the stitches were removed, and no other dressing than the roller bandage applied, and that only for the purpose of support.

On May the 8th he was discharged cured, and at this time he seems to be in as perfect health as before the serious injury sustained. Food and drink by the mouth were permitted on the eighth day after the operation, the food consisting of soup, milk, and other bland substances imposing little or no tax upon the digestive functions of the stomach.

The points of primary interest attaching to this case are the rapidity of healing and the completeness of the cure, the absolute absence of fever after the operation was performed, notwithstanding the gravity and extent of that operation, and finally that an artery of the size of the one severed, should have remained unsecured for sixteen hours, and not have resulted in death from hemorrhage. I infer that the reason this did not result in death must have been due to the great pressure exerted by the effused blood, occasioning by this pressure almost entire occlusion of its lumen.—*Nashville Journal of Medicine and Surgery.*

